# Prognostic value of melatonin-related signature genes in lung adenocarcinoma

**DOI:** 10.1371/journal.pone.0357584

**Published:** 2026-09-15

**Authors:** Hongxia Guo, Lixia Liu, Ying Lu, Yuhui Ma, Xiaolu Ren, Tong Cui

**Affiliations:** 1 Department of Thoracic-cancer, Shanxi Bethune Hospital, Shanxi Academy of Medical Sciences, Tongji Shanxi Hospital, Third Hospital of Shanxi Medical University, Taiyuan, China; 2 Tongji Hospital, Tongji Medical College, Huazhong University of Science and Technology, Wuhan, China; 3 Department of Traditional Chinese Medicine, Shanxi Province Cancer Hospital/Shanxi Hospital Affiliated to Cancer Hospital, Chinese Academy of Medical Sciences/Cancer Hospital Affiliated to Shanxi Medical University, Taiyuan, Shanxi, China; 4 Department of Radiotherapy, Shanxi Province Cancer Hospital/Shanxi Hospital Affiliated to Cancer Hospital, Chinese Academy of Medical Sciences/Cancer Hospital Affiliated to Shanxi Medical University, Taiyuan, Shanxi, China; 5 Department of Radiotherapy, Shanxi Province Cancer Hospital/ Shanxi Hospital Affiliated to Cancer Hospital, Chinese Academy of Medical Sciences/Cancer Hospital Affiliated to Shanxi Medical University, Taiyuan, Shanxi, China; Xiangya Hospital Central South University, CHINA

## Abstract

**Purpose:**

Lung adenocarcinoma (LUAD), the most prevalent lung cancer subtype, has witnessed a dramatic upsurge in frequency over the past 20 years. This study intended to construct a predictive risk model and analyze melatonin (ME)-associated genes in LUAD.

**Methods:**

Differentially expressed genes (DEGs) were found by using DESeq2 to analyze the TCGA-LUAD dataset. ME-DEGs were discovered near the junction, and ME critical module genes were identified by WGCNA. To build a risk model, model genes from ME-DEGs were chosen using univariate and multivariate Cox analysis. The samples were categorized as either low-risk or high-risk. The model was verified using the GSE31210 dataset. Gene expression was confirmed by RT-qPCR, immunological studies were performed, and a nomogram was developed.

**Results:**

The 98 ME-DEGs were obtained by combining 1,052 ME key module genes with 5,449 LUAD-DEGs. Eight model genes were selected using univariate and multivariate cox regression analyses. The risk model, which was constructed using model genes, showed good predictive performance in both the TCGA-LUAD and GSE31210 datasets. Additionally, the nomogram’s superior predictive accuracy for LUAD was confirmed by calibration and receiver operating characteristic (ROC) curves. Additionally, the results of immune analysis showed that ALG3 and FRY had significant relationships with immune cells and immune checkpoints, and that E2F1 had a significant negative association with FRY.

**Conclusion:**

We developed and validated a novel ME-related prognostic model for LUAD. This algorithm may be able to predict patient outcomes and provide recommendations for tailored immunotherapy.

## Introduction

Lung cancer (LC) ranks as the primary cause of cancer – related deaths both globally and in China [1]. Non-small cell lung cancer (NSCLC) constitutes over 85% of all lung cancer cases, among which lung adenocarcinoma (LUAD) represents the most prevalent [2]. The incidence of LUAD has exhibited a significant upward trend over the past two decades. Despite the emergence of targeted therapy and the wide use of checkpoint inhibitors, the 5-year overall survival (OS) rate for LC remains below 20% [3,4]. Moreover, LUAD, as a highly invasive disease, has a poor prognosis and its potential molecular mechanisms remain unclear [5].

Melatonin (ME) is regarded as the most vital regulator of the circadian rhythm. The pineal gland functions as the primary producer of ME within the body [6]. However, numerous studies have validated that other tissues and cells such as the skin, gastrointestinal tract, bone marrow, and lymphocytes are also capable of secreting ME at relatively low levels. Although the regulation of the circadian rhythm is the most well – recognized function of ME secretion, research has shown that it might affect several other biochemical and immunological pathways in the body [7–9]. ME is renowned for its potent antioxidant capabilities [10]. It has the ability to counteract free radicals like reactive oxygen species, nitric oxide, and other substances. In addition, it can upregulate the expression and enhance the activity of antioxidant peptides and enzymes, for instance, glutathione, glutathione peroxidase, superoxide dismutase, catalase, and glutathione reductase [11]. The concentration of ME fluctuates between day and night [12]. An increase in ME release, whether during the day or at night, can result in a decrease in oxidative stress and markers associated with genomic instability [13].

ME exhibits an intriguing characteristic in its immunoregulatory function. Evidently, it impacts the proliferation and functionality of immune cells [14]. It has been proven that the ablation of the pineal gland not only disturbs the circadian rhythm but also causes a substantial reduction in the frequency and activity levels of immune cells [15]. The supplementation of ME can boost the proliferation of neutrophils and monocytes and improve the phagocytic capacity of these cells [16]. Additionally, research has shown that the introduction of ME into the cerebral region can increase the activity of natural killer (NK) cells [17]. T lymphocytes, which play a vital role as immune cells in cancer therapy, can also be influenced by ME. These cells possess high-affinity binding sites for ME [18]. ME not only has important antioxidant properties and could control the immune system [19], but also could protect healthy cells from carcinogens and inhibit the development, invasion, metastasis and angiogenesis of various malignant tumors [20]. The anticancer activity of ME has been verified in various tumors, including breast cancer, liver cancer, colon cancer, leukemia and pancreatic cancer [21]. Furthermore, studies have shown that ME inhibits angiogenesis and lymphangiogenesis in LUAD by regulating macrophages and the NLRP3 inflammasome in the tumor microenvironment, indicating that it may be a potential candidate for LUAD management [22]. Meanwhile, ME suppresses the proliferation, migration, and invasion of LUAD cells by modulating the circRNA-miRNA-TOX3 pathway, demonstrating its potential in inhibiting LUAD progression [23]. However, the specific regulatory mechanism of ME in LUAD remains unclear. Therefore, it is particularly important to further investigate the potential of ME-related genes (ME-RGs) as biomarkers for LUAD, and to uncover their molecular mechanisms and prognostic characteristics.

In this research, through the implementation of a comprehensive array of bioinformatics analyses on ME-RGs, our objective is to construct an innovative predictive risk model. We will also explore the prognostic significance of ME-RGs in LUAD and the potential molecular regulatory pathways they are involved in. The findings of this study are expected to offer novel insights and references for the clinical diagnosis and treatment strategies of LUAD, thereby contributing to the advancement of LUAD management in the medical field.

## Materials and methods

### Data source

The TCGA (https://portal.gdc.cancer.gov/) database supplied the transcriptome data, patient profiles, and survival data for the LUAD. A total of 539 LUAD samples and 59 normal lung tissue samples were included in the TCGA-LUAD cohort and used as the training set. The GSE31210 dataset from Gene Expression Omnibus (GEO) (https://www.ncbi.nlm.nih.gov/) contains 226 LUAD samples and 20 normal lung tissue samples. It encompasses the messenger ribonucleic acid (mRNA) expression profiles and survival information based on the GPL570 sequencing platform, and was used as the validation set. Twelve ME - RGs were obtained from the literature [24–29] (S1 Table).

### Analysis of functional annotations and correlations between genes related to ME

We used the Gene Ontology (GO) and Kyoto Encyclopedia of Genes and Genomes (KEGG) pathway enrichment analyses with the clusterProfiler software (Version 3.14.3) to investigate the functional annotation of ME-RGs. The statistical significance for these enrichments was set at an adjusted p-value (p.adj) < 0.05 [30]. After obtaining the enrichment results, we used the ggplot2 R package (version 3.3.2) to generate chord diagrams, which visually represent the relationships between genes and enriched pathways [31]. To investigate correlations among ME-RGs, Spearman correlation analysis was performed on the TCGA-LUAD dataset, and a heatmap of the correlation coefficient matrix was generated.

### Acquisition of Key ME Module Genes via Weighted Gene Co-expression Network Analysis (WGCNA)

The GSVA program (version 1.7-9) was used to determine the ME scores for each sample in the TCGA-LUAD dataset based on 12 ME-RGs [32]. Subsequently, using ME score as the trait, we further screened the modules most highly correlated with ME score and the genes within them. Firstly, WGCNA (version 3.4.3) was used to perform hierarchical clustering on all samples in the TCGA-LUAD dataset, and any exceptional samples were then removed [33]. Then, the ideal soft threshold (β) was established by assessing the degree to which the network conformed to a scale-free distribution. Next, the adjacency and similarity of genes were utilized to construct a hierarchical clustering dendrogram of genes. The modules were merged using the dynamic tree-cutting algorithm, with a minimum of 100 genes per module. Finally, an evaluation of the relationship between each module and the ME scores was conducted. Then, among the modules that showed the strongest correlation with the ME scores (p < 0.05), the essential genes of the ME modules were extracted (p < 0.05).

### Identification of differentially expressed genes (DEGs) in LUAD

DEGs in the TCGA-LUAD dataset were identified using the DESeq2 tool (version 1.34.0), with the criterion set to Log2 fold change (|Log2FC|) > 1 and *p*.adj < 0.05, allowing for the comparison of gene expression variations between LUAD and normal samples [34]. Volcano plots were created using the ggplot2 package (version 3.3.2), and heatmaps were generated with the ComplexHeatmap package (version 2.14.0) [31,35].

### ME-DEGs Identification and Functional Annotation Analysis

After that, ME-DEGs were discovered by utilizing the VennDiagram R program (version 1.7.1) to determine the intersection between LUAD-DEGs and the ME key module genes [36]. To explore the functional annotation of ME-DEGs, we conducted GO and KEGG pathway enrichment analyses using the clusterProfiler package (version 3.14.3), with the statistical significance of these enrichments set as an p.adj < 0.05 [30].

### Univariate and multivariate cox analysis

Univariate Cox regression analysis was conducted on the ME-DEGs with available survival data from the TCGA-LUAD dataset. Results with a p-value < 0.2 were considered statistically significant. To refine the set of potential model genes, multivariate Cox regression analysis was performed using the step() function with the parameter direction set to “both,” which optimized the multivariate regression model and identified the final model genes.

### Risk model construction and evaluation

The evaluation of model gene expressions and regression coefficients obtained from the multivariate Cox analysis served as the foundation for the creation of the prognostic risk model. The risk score calculation formula is as follows:


$$\begin{array}{c} \text{Riskscore }=\sum_{\text{i }=\text{1}}^{\text{n}}\text{coef}({\text{gene}}_{\text{i}})*\text{expr}({\text{gene}}_{\text{i}}) \end{array}$$

The expr represents the degree of gene expression, while the coef represents each gene’s risk coefficient. Using the direction function, the risk scores for 539 LUAD samples in the TCGA-LUAD dataset were then calculated. The risk score median was then used as a reference to split all samples into high- and low-risk groups. After the risk scores were ranked, a risk score curve was created. Furthermore, two risk groups’ scatter plots were employed to evaluate the prediction power of the risk model. Next, two distinct risk categories’ levels of model gene expression were shown on the heat map. Moreover, two risk categories were subjected to a Kaplan-Meier (K-M) survival analysis using the R package survival (version 3.2−3) [37]. The Survival receiver operating characteristic (ROC) (version 1.0.3) was used to generate ROC curves for survival durations of 1, 3, and 5 years. The appropriate area under the curve (AUC) values were then calculated [38]. The risk model was subsequently validated on the GSE31210 dataset using the same methodology.

### Independent prognostic evaluation and development of nomograms

The TCGA-LUAD dataset was utilized to analyze the disparities in risk scores and the distribution of model genes among different risk groups across 7 clinical characteristics samples of LUAD patients, including gender, race, age, stage, T, M, and N stage. Subsequently, these seven clinical traits and risk scores were assessed for independent prognostic value using univariate and multivariate cox analysis (HR ≠ 1, p < 0.05). A nomogram based on independent prognostic factors was created using the rms program (version 6.0−1) to estimate survival rates for patients with LUAD at 1, 3, and 5 years [39]. ROC and calibration curves were used to evaluat the nomogram’s accuracy and dependability.

### Gene set enrichment analysis (GSEA) and validation of model genes expression

Next, GSEA was performed on different risk categories within the TCGA-LUAD dataset. First, DEGs between high- and low-risk groups were identified using the DESeq2 tool (version 1.34.0) [34] and ranked based on log_2_FC values (|Log_2_FC| > 0.5, *p*.adj < 0.05). Following this, the clusterProfiler package (version 4.7.1) [30] was employed for analysis, utilizing KEGG gene sets as enrichment background (p < 0.05, q < 0.25). To show the different expression of model genes between the normal and LUAD groups, a box plot was utilized.

### Assessment of immune cell infiltration across various risk categories

To investigate immune cell infiltration across all samples in the TCGA-LUAD dataset, the CIBERSORT algorithm was applied to analyze immune cell composition in high- and low-risk group samples, evaluating the proportion of different immune cell types within each sample. Subsequently, the Wilcoxon signed-rank test was used to assess differences in immune cell composition between the high- and low-risk groups (p < 0.05). Additionally, the Spearman technique was used to calculate the connections between model genes and various immune cells as well as between immune cells. Meanwhile, the expression differences of 39 immunological checkpoints in various risk groups were depicted using a box plot (p < 0.05) [40]. The correlation between various immune checkpoints and model genes was assessed.

### The mutant landscape of model genes

The somatic mutation data, CNV data, and methylation data were acquired from the TCGA-LUAD dataset. The mutations of model genes were analyzed utilizing the maftools software. The CNV status of model genes was examined and a comparative investigation was performed on the variation in methylation levels between model genes in LUAD and normal samples (*p* < 0.05).

### Drug sensitivity analysis for chemotherapy

To investigate differences in drug sensitivity between high- and low-risk patient groups, the pRRophetic software (version 0.5) was used to determine the half maximum inhibitory concentration (IC50) values of 138 drugs for each tumor sample in both risk categories in the TCGA-LUAD dataset [41]. The Wilcox test method (p < 0.05) was used to assess the variation in sensitivity levels of 138 drugs across various risk categories in the TCGA-LUAD dataset.

### Reverse Transcription-Quantitative Polymerase Chain Reaction (RT-qPCR)

To validate the findings from the analysis of the public database, we purchased a total of nine cell samples. Specifically, there were three A549/DDP cell samples (obtained from Changsha Abiowell Biotechnology Co., LTD), three Calu-3 cell samples (also from Changsha Abiowell Biotechnology Co., LTD), and three BEAS-2B cell samples (Changsha Abiowell Biotechnology Co., LTD). During the experimental procedures, for RNA extraction, we strictly followed the manufacturer’s instructions and used TRIzol (produced by Ambion, Austin, USA). Subsequently, cDNA was synthesized according to the instructions of the First Strand cDNA Synthesis Kit from Servicebio (Wuhan, China). After that, qPCR was performed using the 2x Universal Blue SYBR Green qPCR Master Mix from the same company (Servicebio, Wuhan, China) in line with the provided manual. The primer sequences for PCR are listed in S2 Table. GAPDH was used as the internal reference gene, and the 2-ΔΔCt method was applied to detect and calculate the gene expression levels [42].

### Analytical statistics

R (version 4.1.0) was used to conduct the statistical study. The differentially expressed analysis tool used was DESeq2.

## Results

### Correlation analysis and functional analysis of ME-RGs in LUAD

**Fig 1:**
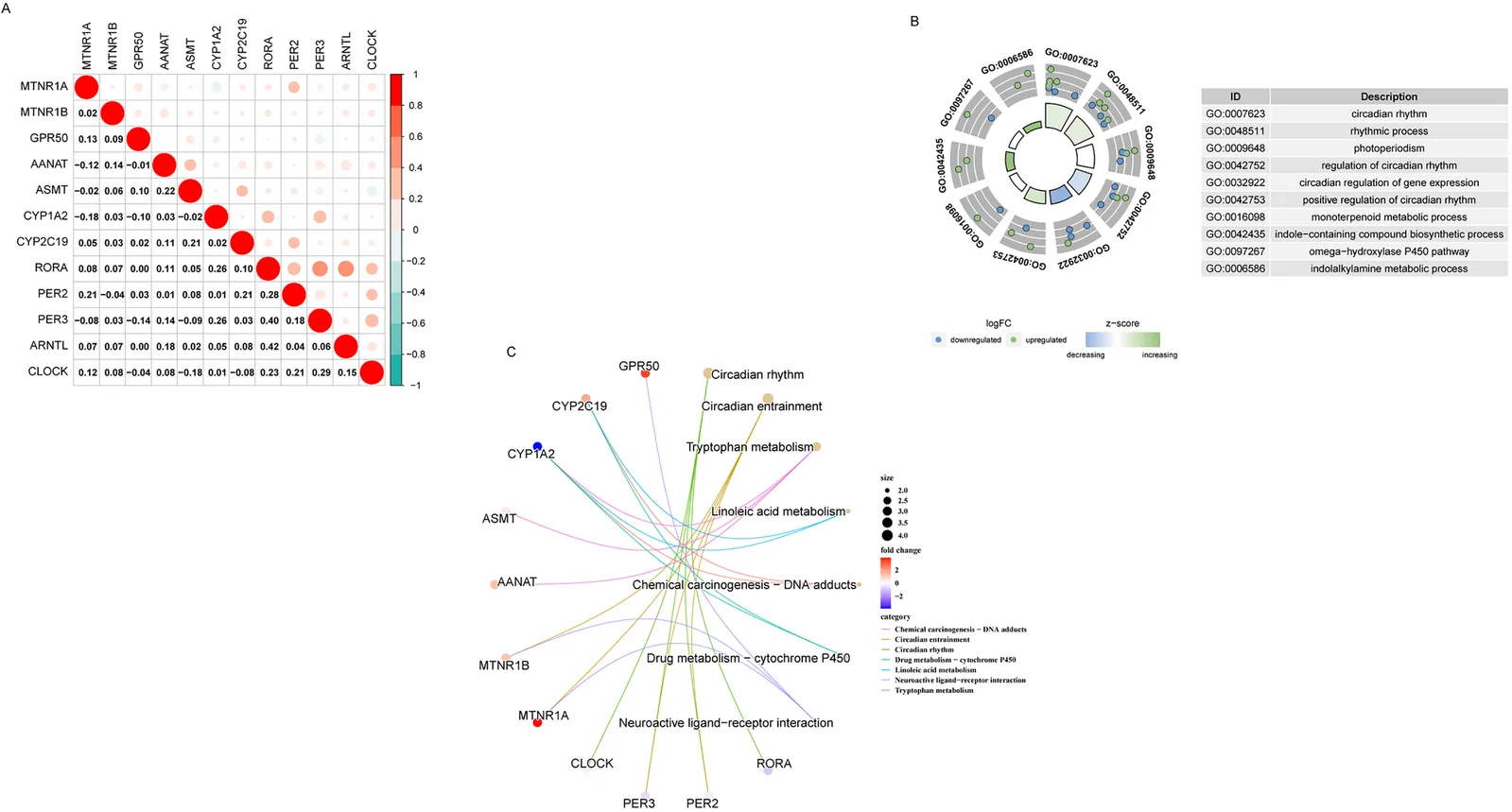
Analysis of the correlation between ME-RGs in LUAD and functional enrichment analysis of ME-RGs. (A) Correlation heatmap of ME-RGs. (B-C) GO and KEGG enrichment analysis.

The correlation heatmap of ME-RGs showed that RORA exhibited the strongest positive correlations with AINTL (R = 0.42) and PER3 (R = 0.40) (Fig 1A). The KEGG pathways were enriched in circadian rhythm, circadian entrainment, and tryptophan metabolism, whereas the activities of ME-RGs were significantly enriched in circadian rhythm, rhythmic process, and photoperiodism, according to GO enrichment analysis (Fig 1B-C).

### Acquisition of ME-DEGs as well as functional annotation analysis

**Fig 2:**
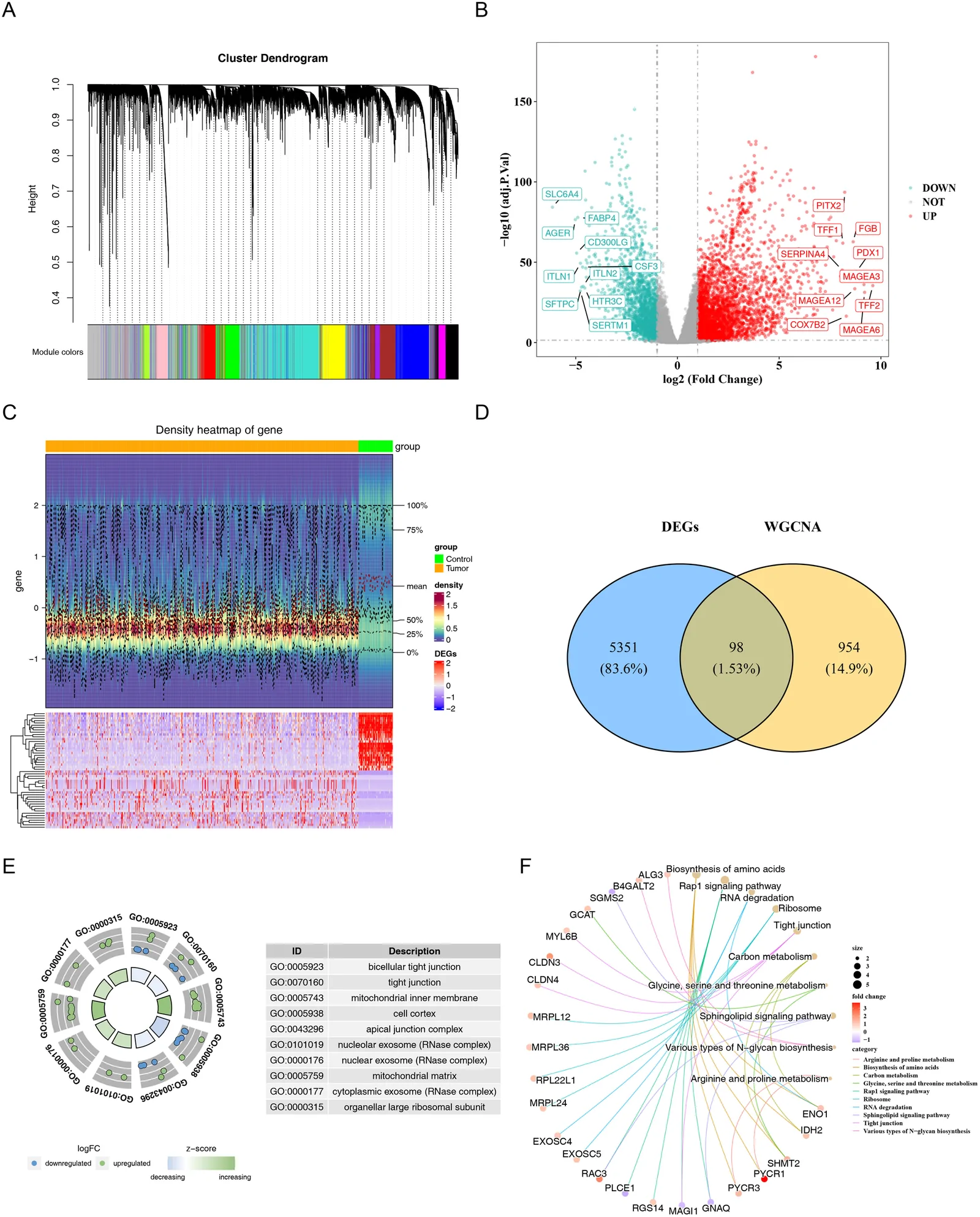
Acquisition of ME-DEGs and functional annotation analysis. (A) The module clustering dendrogram of genes. (B) DEGs between LUAD and health samples in a volcano plot. (C) Heatmap of Top 50 DEGs. (D) Identification of differential genes related to ME-DEGs. (E-F) GO and KEGG enrichment analysis of differential genes related to ME-DEGs.

Subsequently, WGCNA was performed using the ME score derived from ME-RGs calculations as a trait. The results obtained from the analysis of sample clustering indicated the absence of any exceptional samples in TCGA-LUAD dataset (S1 Fig. A). Using a scale-free network architecture and a threshold value of 0.85 for the scale-free R2, the soft threshold β was chosen, and the ideal value was determined to be 7 (S1 Fig. B). Twelve gene modules were built using the hybrid dynamic tree cutting algorithm’s specifications (Fig 2A). The yellow module was then identified as a key module based on the correlation with the ME score (*p* = 7 × 10^−57^, Cor = −0.59), and 1,052 ME key module genes were recovered (S1 Fig. C). Although Cor = −0.59 indicates a moderate correlation, such associations between individual molecular pathways and the global transcriptome are expected in complex biological systems. More importantly, this correlation demonstrates extremely high statistical significance (with a very low p-value). Therefore, despite the moderate correlation, the screened genes provide a solid foundation for constructing a reliable prognostic model. When comparing LUAD samples to normal samples, 5,449 LUAD-DEGs were found in the TCGA-LUAD dataset. Of these, 1,918 LUAD-DEGs exhibited downregulation while 3,531 LUAD-DEGs were shown to be upregulated in LUAD samples. Sorting by |log2FC| revealed that the heat map displayed the top 50 LUAD-DEGs, while the volcano map displayed the top 10 (Fig 2B-C). Ultimately, 98 ME-DEGs were identified as the intersection of 1,052 ME essential module genes and 5,449 LUAD-DEGs (Fig 2D). According to the functional enrichment analysis of 98 ME-DEGs, the KEGG pathways were enriched in RNA degradation, rap1 signaling pathway, and amino acid biosynthesis, while the functions were considerably enriched in bicellular tight junction, tight junction, and mitochondrial inner membrane (Fig 2E-F).

### Construction and identification of risk model

**Fig 3:**
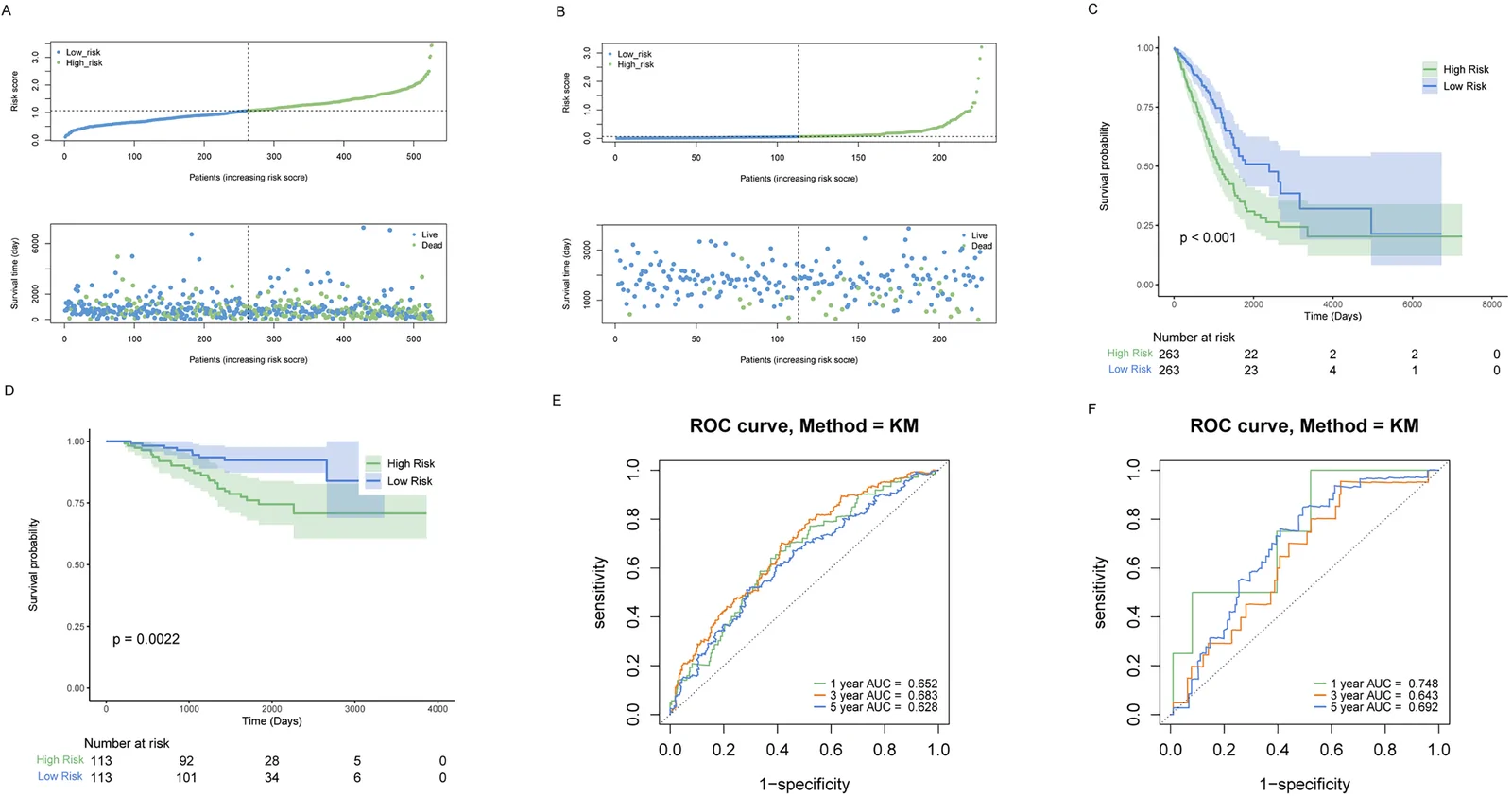
Prognostic evaluation of the risk model in TCGA-LUAD and GSE31210 datasets. (A-B) Risk curve of risk groups in TCGA-LUAD and GSE31210 datasets. (C-D) Kaplan-Meier survival curves of TCGA-LUAD and GSE31210 datasets. (E-F) ROC curve of TCGA-LUAD and GSE31210 datasets.

Following the examination of 98 ME-DEGs, 48 ME-DEGs associated with prognosis were discovered in the TCGA-LUAD dataset by univariate Cox regression analysis (*p* < 0.2) (S2 Fig. A). Subsequently, multivariate Cox regression analysis was employed to discover 8 model genes (*BOP1*, *ALG3*, *FRY*, *MTMR10*, *HAGHL*, *SCN1A*, *TMEM52* and *HSPB9*) from 48 ME-DEGs related to prognosis and construct a risk model (S2 Fig. B). The samples were classified into high and low-risk groups by calculating risk scores with the direction function and using the median as a threshold. The heat maps illustrated the level of gene expression in two distinct risk groups across both the TCGA-LUAD and GSE31210 datasets (S2 Fig. C). As the risk ratings in the samples increased, the scatter plot showed a discernible increase in the number of fatalities (Fig 3A-B). The K-M curves showed that the low-risk group of LUAD patients had a longer survival period (Fig 3C-D). The risk model was successful in forecasting results at 1, 3, and 5 years, as demonstrated by the ROC curves, which showed that its AUC values were higher than 0.6 (Fig 3E-F). Although the model’s discriminatory power is moderate, this is not uncommon among multi-gene prognostic models based on transcriptomic data, particularly in highly heterogeneous cancers such as LUAD [43]. The effectiveness of the risk model was validated through analysis of the GSE31210 dataset.

### Nomogram demonstrated the ability to make correct predictions

**Fig 4:**
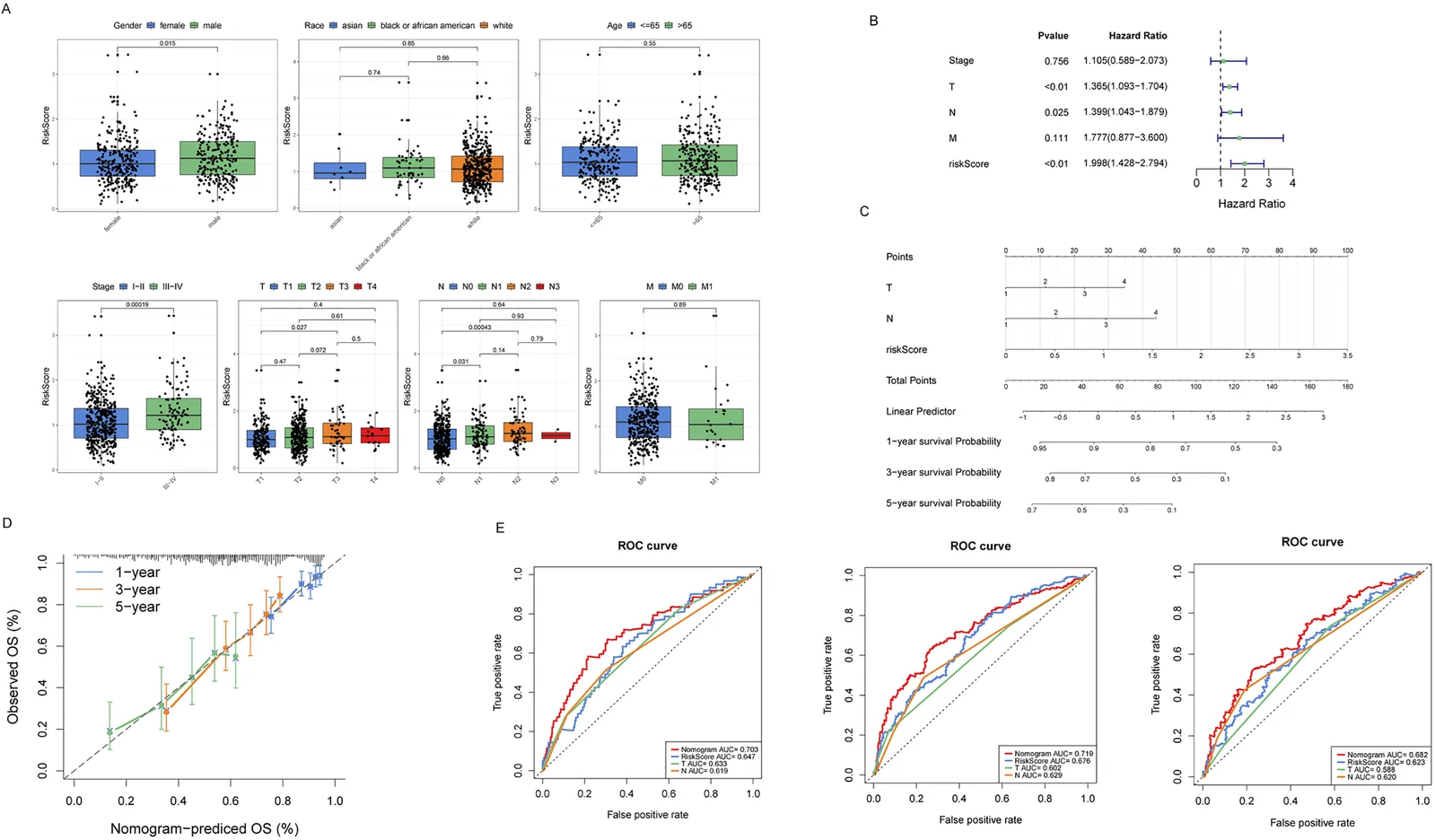
Nomogram exhibited an accurate predictive capability. (A) Variations in Risk Scores for Patients with Various Clinical Features. (B) Multivariate Cox analysis forest plot. (C) The nomogram model for predicting the OS of patients from TCGA-LUAD. (D) Nomogram calibration curve for OS prediction. (E) Nomogram’s ROC curve.

Next, clinical characteristic analyses were conducted in the high-risk and low-risk groups. The heat maps illustrated the levels of model gene expressions in two distinct risk groups across 7 clinical features (gender, race, age, stage, T, M, and N stage) (S3 Fig. A). The boxplot results revealed significant disparities in risk scores between Gender and Stage (Fig 4A). Subsequently, an independent prognostic analysis was conducted. Overall survival (OS) in LUAD was found to be influenced by risk score, stage, and TMN stage, according to univariate cox regression analysis (S3 Fig. B). Through PH hypothesis testing and multivariate Cox analysis, risk score and TN staging were further identified as independent prognostic factors, leading to the development of a nomogram (Fig 4B-C). The nomogram demonstrated good prediction performance; the AUC values for each of these time periods were more than 0.6, and the survival projections for 1, 3, and 5 years closely matched the theoretical straight line (Fig 4D-E).

### Different risk groups’ DEGs were more abundant in pathways linked to tumors and the immune system

Significant enrichments in immunological and tumor-related pathways, such as systemic lupus erythematosus, olfactory transduction, cell cycle, p53 signaling pathway, and taste transduction, were found in the GSEA enrichment analysis results of DEGs in two risk groups (**S4 Fig**.).

### The associations of model genes with immune cells and immune checkpoints

**Fig 5:**
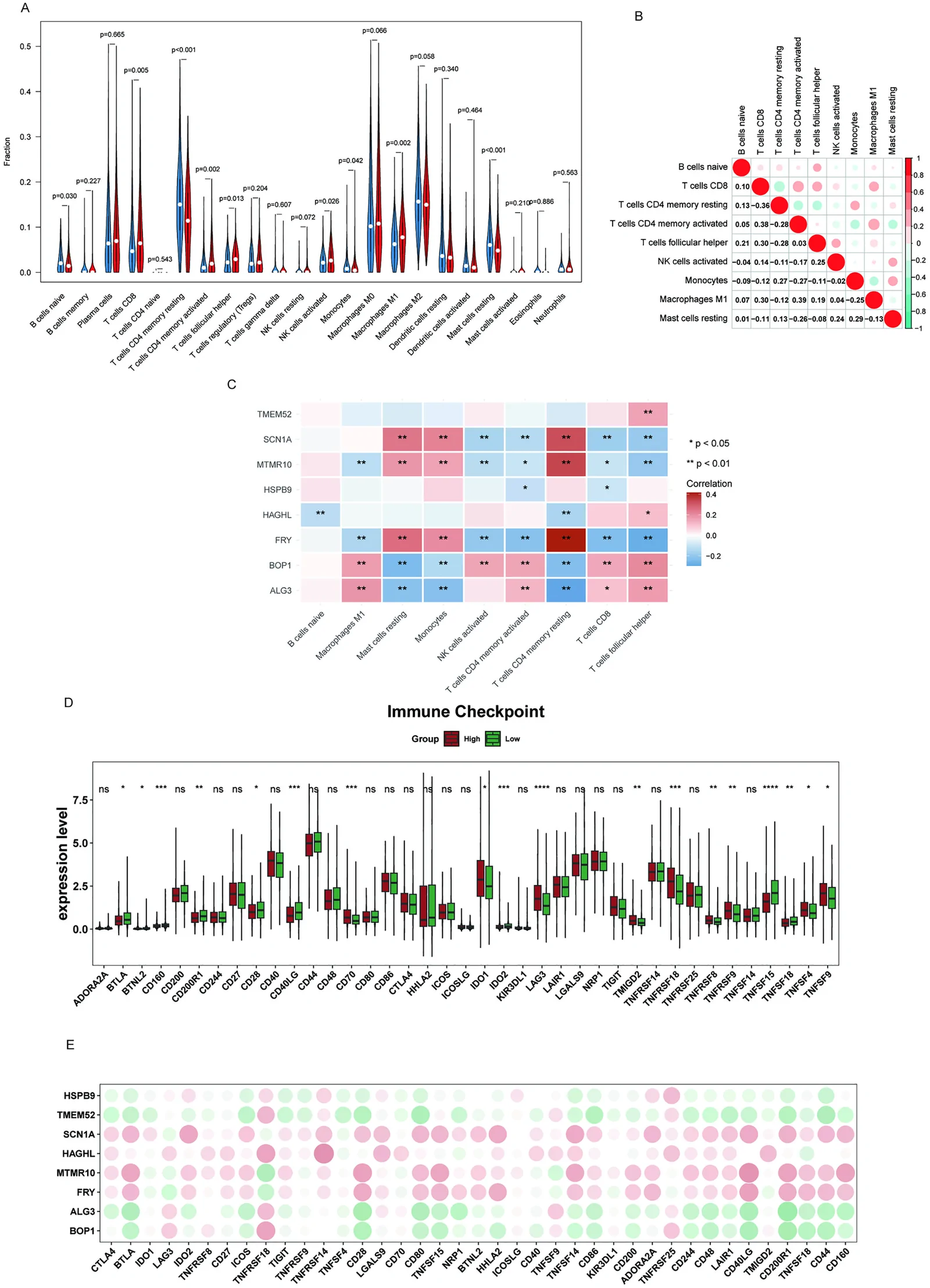
Relationships between model genes and immunological checkpoints and cells. (A) The percentage of immune cells in high- and low-risk groups. (B) Differentially infiltrated immune cells’ correlation. (C) Analysis of the relationship between immune cells and prognostic genes. (D) The expression status of immune checkpoints in different risk groups. (E) Correlation analysis between eight prognostic genes and immune checkpoints.

To understand the immune cell infiltration patterns in the high- and low-risk groups, relevant analyses were conducted. The Wilcox test identified notable disparities in the prevalence of nine immune cell categories (naive B cells, resting mast cells, CD8 + T cells, activated NK cells, etc.) in two risk groups (Fig 5A). The Spearman correlation analysis revealed a strong positive correlation. Specifically, a significant positive association was observed between resting memory CD4 + T cells and FRY, with an R value of 0.41. Additionally, there was a notable positive relationship between activated memory CD4 + T cells and M1 macrophages, and the R value for this correlation was 0.39. Furthermore, a substantial inverse relationship between ALG3 and resting memory CD4 + T cells was found (R = −0.30) (Fig 5B-C). Additionally, the expression levels of 18 immunological checkpoints (BTLA, BTNL2, CD160, CD200R1, CD28, CD40LG, CD70, IDO1, IDO2, LAG3, TMIGD2, TNFRSF18, TNFRSF8, TNFRSF9, TNFSF15, TNFSF18, TNFSF9) showed significant differences between two risk groups (Fig 5D). Notably, ALG3 and BTLA, CD28, TNFSF15, CD40LG, and CD200R1 were found to be negatively correlated by the Spearman correlation analysis, while FRY and these same factors were identified as positively correlated (Fig 5E). Moreover, chemotherapeutic drug sensitivity analysis results suggested that IC_50_ of 41 drugs differed markedly among risk groups, such as A.443654, A.770041 and CCT007093 (S5 Fig. A**-B**).

### The methylation levels of *BOP1*, *ALG3*, *HSPB9*, *FRY* and *MTMR10* exhibited an opposite trend with the expression levels in LUAD

**Fig 6:**
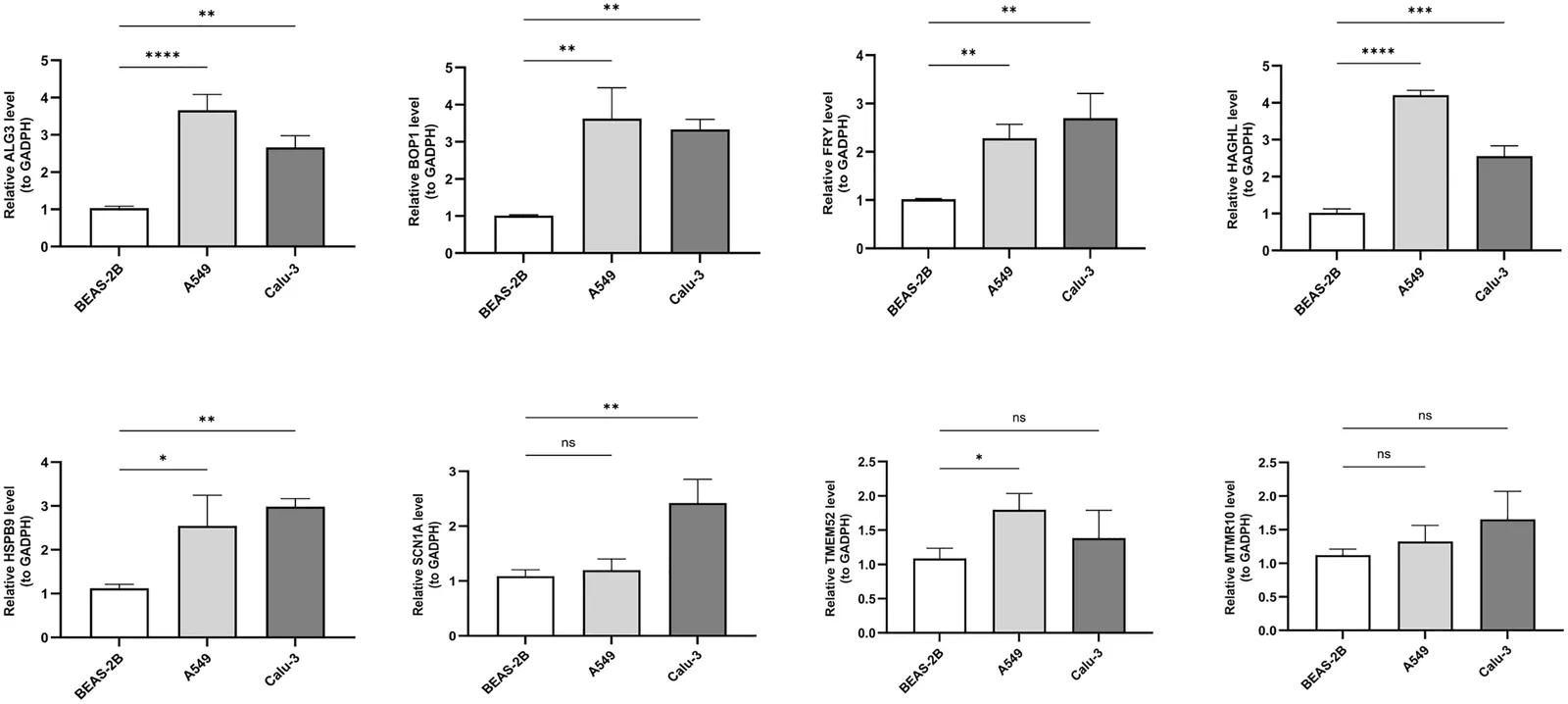
The expression of *BOP1*, *ALG3*, *HSPB9* and *HAGHL* in A549/DDP cells and Calu-3 cells.

The analysis of the mutations and the CNV status of model genes was performed (**S6 Fig**.). The methylation levels of model genes were also assessed in LUAD and normal samples. In contrast to normal samples, the methylation levels of FRY and MTMR10 showed a large rise in LUAD, while the methylation levels of BOP1, ALG3, and HSPB9 were significantly reduced (S7 Fig. A). The box plot results in the TCGA-LUAD dataset revealed significant up-regulation of *BOP1*, *ALG3*, *HAGHL*, *TMEM52*, and *HSPB9* gene expressions in LUAD. Conversely, *FRY*, *MTMR10*, and *SCN1A* gene expressions were significantly down-regulated in LUAD (S7 Fig. B). Importantly, in RT-qPCR, *BOP1*, *ALG3*, *HSPB9* and *HAGHL* were also significantly up-regulated in A549/DDP cells and Calu-3 cells (Fig 6). This finding was consistent with the results obtained from bioinformatics analysis.

## Discussion

Global health is heavily burdened by the constantly increasing prevalence of LUAD, the most prevalent form of lung cancer. Despite advances in modern medicine’s approach to cancer therapy, LUAD patients have a persistently low 5-year survival rate. One of the main problems is the lack of accurate prognostic evaluation tools and early detection [44]. By investigating the significance of genes linked to ME in LUAD, this study developed a predictive model associated with ME through a number of analyses, offering fresh perspectives for enhancing the prognosis evaluation and care of LUAD patients.

In this study, through comprehensive analysis of the TCGA – LUAD dataset, we successfully identified eight prognostic genes related to ME (BOP1, ALG3, FRY, MTMR10, HAGHL, SCN1A, TMEM52, and HSPB9). As far as we know, the prognostic significance of these eight genes in LUAD remains incompletely understood, with a dearth of comprehensive information available, particularly with regard to ME regulation [45]. This study aimed to reveal their potential role in LUAD. Although few reports on the association between these genes and ME in LUAD have been retrieved so far, some of these genes have been found to have certain biological functions in other diseases. In certain malignancies, for instance, ALG3 has been linked to glycosylation and cell proliferation; this suggests that its aberrant expression in LUAD may also be implicated in the control of tumor cell biological activities [46]. According to the ROC analysis results, the prognostic model built using these eight genes may be useful in forecasting LUAD patients’ probabilities of survival. AUC scores for 1-, 3-, and 5-year predictions were more than 0.6, indicating that the model has reasonable accuracy. This result demonstrates some competitiveness when compared to other prognostic models developed in related studies and offers additional prospective biomarkers for the early diagnosis and prognosis evaluation of LUAD.

Immunoanalysis findings showed that patients with high- and low-risk LUAD had notable variations in immune cell infiltration and immune checkpoint expression. Regarding immune cells, M1 macrophages and activated memory CD4 + T cells showed a positive correlation, indicating that immune cell synergy may be crucial for the development of LUAD [47]. Resting memory CD4 ⁺ T cells showed a negative correlation with ALG3 but a positive correlation with FRY—an opposing pattern indicating that ALG3 and FRY may exert contrasting effects on immune cell function. Regarding immune checkpoints, ALG3 and FRY exhibited opposite correlations with multiple checkpoints (e.g., BTLA, CD28), further supporting their distinct roles in immune regulation. Specifically, ALG3 is a key enzyme involved in protein N-linked glycosylation [48]. Recent studies have shown that abnormal glycosylation modifications in tumor cells can directly regulate the stability, localization, and function of immune checkpoint proteins (e.g., PD-L1) [49]. Therefore, we hypothesize that in LUAD, high expression of ALG3 may indirectly affect the activation and function of T cells by altering the glycosylation status of certain key immune regulatory proteins on the surface of tumor cells, thereby shaping an immunosuppressive microenvironment. This is consistent with the correlation we observed between ALG3 and low immune cell infiltration. On the other hand, the FRY protein is a regulator of the Hippo signaling pathway and is involved in the establishment of cell polarity and cell migration [50]. In the tumor microenvironment, disruption of cell polarity and enhanced migratory capacity are typically associated with an invasive phenotype [51]. We hypothesize that downregulation of FRY may disrupt the Hippo pathway, alter the behavior of tumor cells, and release certain chemokines or cytokines to indirectly attract or activate specific immune cell populations, while upregulating immune checkpoints through a feedback mechanism. This association may represent a complex interaction between tumors and the immune system.

The methylation levels of *BOP1*, *ALG3*, *HSPB9*, *FRY*, and *MTMR10* in LUAD patients showed a completely opposite trend to their expression levels. Typically, methylation is linked to the silencing of genes [52]. Consequently, these genes may become upregulated as a result of hypomethylation, which would encourage biological processes linked to tumors such cell division, invasion, and metastasis. Nevertheless, the RT-qPCR verification results for a few genes (including FRY, MTMR10, and SCN1A) in cell samples in this investigation ran counter to the patterns in the database analysis [53]. This may be due to the fact that RNA-seq reflects the overall gene expression trend of the sample, while RT-PCR is a different experimental platform, and there are certain differences. In addition, the relatively small number of samples selected in this experiment may not accurately reflect the expression of these genes in most disease populations, and further large-scale experimental verification is required.

Regarding the role of melatonin in other cancers, several studies have reported interesting findings. Melatonin has been demonstrated to use a variety of ways to suppress cell proliferation and trigger apoptosis in breast cancer [54]. It can modulate the expression of oncogenes and tumor suppressor genes, thereby affecting the growth and survival of breast cancer cells [55]. For example, it may downregulate the expression of certain growth factor receptors, reducing the activation of downstream signaling pathways that promote cell growth [56]. The chemopreventive qualities of melatonin may be attributed to its anti-inflammatory and antioxidant effects in colorectal cancer. It can lessen oxidative stress and scavenge free radicals, both of which are frequently linked to the onset and spread of colorectal cancer [57]. Furthermore, by altering the tumor microenvironment and increasing the sensitivity of cancer cells to chemotherapy, melatonin may improve the effectiveness of chemotherapy medications in the treatment of colorectal cancer, according to some research [58]. In prostate cancer, melatonin has been implicated in regulating the cell cycle and inhibiting angiogenesis [59]. By interfering with the cell cycle progression, it can prevent cancer cells from dividing and multiplying [60]. The anti-angiogenic effect of melatonin may limit the blood supply to the tumor, thereby impeding its growth and metastasis [61]. Although melatonin has many commonalities in cancer research, due to the differences in the biological characteristics, microenvironments and clinical needs of different cancers, this study shows significant differences from other cancer studies at key levels such as genes, microenvironments and clinical applications, providing a theoretical basis for a comprehensive understanding of its anticancer mechanisms and promoting precise applications.

In conclusion, this study aims to improve the prognosis assessment system for LUAD patients by identifying eight ME-related prognostic genes in LUAD and developing a corresponding predictive risk model. For the treatment of LUAD, the findings of immune checkpoint and medication prediction analysis offer possible targets and treatment strategy references. However, this study also has certain limitations. First, the study only used samples from a single source for retrospective analysis and lacked validation with diverse samples. Therefore, future research should expand sample sources and conduct more extensive multi-center clinical studies. Second, although the correlation of gene expression was explored, the mechanisms of gene activation were not analyzed in depth; future studies will further investigate the specific mechanisms of action of these genes in LUAD. Additionally, the current experiments are limited to cell models and have not been validated in clinical samples. Future validation experiments in clinical samples are needed to further evaluate the clinical application potential of these genes.

## Supporting information

S1 Table Melatonin associated genes. (XLSX)

S2 Table The primer sequences used for PCR. (XLSX)

S1 Fig Analysis of data clustering and module-trait associations. (A) Sample clustering to detect outliers. (B) Scale-free soft threshold distribution. (C) Heatmap of module-trait correlations.
(TIF)

S2 Fig Development and delineation of risk model. (A-B) Forest plot of univariate and multivariate Cox results. (C) Heatmap of prognostic gene expression levels.
(TIF)

S3 Fig Association of risk scores with clinical features. (A) The distribution of prognostic genes and various clinical characteristics in groups with high and low risk. (B) Forest plot of univariate Cox analyses.
(TIF)

S4 Fig The results of GSEA. (TIF)

S5 Fig Analysis of sensitive drugs. (A) Analysis of sensitive drugs for high-risk group. (B) Analysis of sensitive drugs for low-risk group.
(TIF)

S6 Fig Mutation and CNV status of prognostic genes. (TIF)

S7 Fig Methylation and expression differences of prognostic genes. (A) LUAD and Control Samples Differ in the Methylation Level of Prognostic Genes. (B) Prognostic gene expression levels in the TCGA-LUAD dataset vary between tumor and control tissues.
(TIF)

## Abbreviations

LC: Lung cancer
NSCLC: Non-small cell lung cancer
LUAD: Lung adenocarcinoma
OS: Overall survival
NK: Natural killer
ME-RGs: Melatonin-related genes
GO: Gene Ontology
KEGG: Kyoto Encyclopedia of Genes and Genomes
DEGs: , Differentially expressed genes
FC: Fold change
ME: Melatonin
WGCNA: Weighted Gene Co-expression Network Analysis
p.adj: Adjusted p-value
AUC: Appropriate area under the curve
GSEA: Gene set enrichment analysis
CNV: Copy number variation
RT-qPCR: Reverse Transcription-Quantitative Polymerase Chain Reaction
